# Serum neurofilament light concentrations are associated with cortical thinning in anorexia nervosa

**DOI:** 10.1017/S0033291723000387

**Published:** 2023-11

**Authors:** Inger Hellerhoff, Fabio Bernardoni, Klaas Bahnsen, Joseph A. King, Arne Doose, Sophie Pauligk, Friederike I. Tam, Merle Mannigel, Katrin Gramatke, Veit Roessner, Katja Akgün, Tjalf Ziemssen, Stefan Ehrlich

**Affiliations:** 1 Division of Psychological and Social Medicine and Developmental Neurosciences, Translational Developmental Neuroscience Section, Faculty of Medicine, Technische Universität Dresden, Dresden, Germany; 2 Department of Child and Adolescent Psychiatry, Eating Disorder Research and Treatment Center, Faculty of Medicine, Technische Universität Dresden, Dresden, Germany; 3 Department of Child and Adolescent Psychiatry, Faculty of Medicine, University Hospital Carl Gustav Carus, Technische Universität Dresden, Dresden, Germany; 4 Center of Clinical Neuroscience, Neurological Clinic, Faculty of Medicine, University Hospital Carl Gustav Carus, Technische Universität Dresden, Dresden, Germany

**Keywords:** Anorexia nervosa, cortical thickness, glial fibrillary acidic protein, neurofilament light, tau protein

## Abstract

**Background:**

Anorexia nervosa (AN) is characterized by severe emaciation and drastic reductions of brain mass, but the underlying mechanisms remain unclear. The present study investigated the putative association between the serum-based protein markers of brain damage neurofilament light (NF-L), tau protein, and glial fibrillary acidic protein (GFAP) and cortical thinning in acute AN.

**Methods:**

Blood samples and magnetic resonance imaging scans were obtained from 52 predominantly adolescent, female patients with AN before and after partial weight restoration (increase in body mass index >14%). The effect of marker levels before weight gain and change in marker levels on cortical thickness (CT) was modeled at each vertex of the cortical surface using linear mixed-effect models. To test whether the observed effects were specific to AN, follow-up analyses exploring a potential general association of marker levels with CT were conducted in a female healthy control (HC) sample (*n* = 147).

**Results:**

In AN, higher baseline levels of NF-L, an established marker of axonal damage, were associated with lower CT in several regions, with the most prominent clusters located in bilateral temporal lobes. Tau protein and GFAP were not associated with CT. In HC, no associations between damage marker levels and CT were detected.

**Conclusions:**

A speculative interpretation would be that cortical thinning in acute AN might be at least partially a result of axonal damage processes. Further studies should thus test the potential of serum NF-L to become a reliable, low-cost and minimally invasive marker of structural brain alterations in AN.

## Introduction

Anorexia nervosa (AN) is characterized by a persistent restriction in energy intake leading to cachexia and serious medical complications (Voderholzer, Haas, Correll, & Körner, 2020; Westmoreland, Krantz, & Mehler, 2016). Furthermore, acutely underweight patients with AN show markedly reduced brain mass, which can often be observed on computed tomography scans or magnetic resonance imaging (MRI) scans even at an individual level (King, Frank, Thompson, & Ehrlich, 2018; Nussbaum, Shenker, Marc, & Klein, 1980; Seitz et al., 2014).

An established measure for structural brain changes is cortical thickness (CT). Several studies have shown widespread CT reductions in acutely underweight AN patients (Bernardoni et al., 2016; Walton et al., 2022). Most of these alterations seem to be reversible upon weight gain/recovery (Bernardoni et al., 2016; Castro-Fornieles et al., 2021), and an increase in body mass index (BMI) has been shown to be associated with increase in CT (Bernardoni et al., 2016). However, the mechanisms underlying these alterations are still unknown. Post-mortem studies suggest the hypothesis of alterations in size and/or morphology of glia cells or neurons but are very limited in number and sample size (Martin, 1958; Neumärker et al., 1997).

To test this hypothesis with in-vivo measurements, several studies have therefore investigated blood markers of brain damage (Doose et al., 2021; Hellerhoff et al., 2021; Nilsson et al., 2019; Wentz et al., 2021). These markers provide information about specific processes on a cellular level (Zetterberg, Smith, & Blennow, 2013). The most studied marker in this context is neurofilament light (NF-L), a structural scaffolding protein expressed in neurons (Khalil et al., 2018). Upon axonal injury, increasing concentrations of NF-L are found in cerebrospinal fluid (CSF) and blood (Gaetani et al., 2019). Interestingly, this was also reported in patients with acute AN in two independent studies (Hellerhoff et al., 2021; Nilsson et al., 2019). Furthermore, during weight restoration treatment, an increase in BMI was related to decreasing NF-L levels (Hellerhoff et al., 2021). Another marker for axonal injury is tau protein, a microtubule-binding protein expressed in great amounts in thin non-myelinated axons of cortical interneurons (Kawata et al., 2016; Randall et al., 2013; Trojanowski, Schuck, Schmidt, & Lee, 1989; Zetterberg et al., 2013). One recent study found increased serum tau concentrations in patients with acute AN both before and after short-term partial weight restoration (Hellerhoff et al., 2021) a result that partly matches the NF-L findings but still needs replication.

Not only neurons but also glial cells might be affected in the acutely underweight state of AN. Elevated levels of glial fibrillary acidic protein (GFAP), an intermediate filament found in mature astrocytes (Eng, Ghirnikar, & Lee, 2000), have been associated with astroglial injury (Aurell et al., 1991). While Ehrlich et al. (2008) found no difference in GFAP levels between patients with AN and healthy control participants (HC), a more recent study found increased GFAP levels in acutely underweight AN patients, which were not detectable anymore after partial weight restoration (Hellerhoff et al., 2021). These inconsistencies regarding GFAP levels in AN might be due to differences in laboratory methods: Recent studies have profited from the use of single-molecule array (Simoa™) technology, reaching significantly lower limits of detection in comparison to conventional enzyme-linked immunosorbent assays (Ehrlich et al., 2008; Kuhle et al., 2016; Quanterix, 2013, 2017; Wilson et al., 2016). Importantly, this new technology has also facilitated the detection of even slight elevations in peripheral blood samples for both NF-L and tau protein (Kuhle et al., 2016; Quanterix, 2013).

The aforementioned studies suggest neuronal and astroglial alterations/damage processes occurring in acute AN, which could contribute to the brain morphological changes seen on MRI scans. Studies in healthy populations (Khalil et al., 2020; Rajan et al., 2020; Vågberg et al., 2015) as well as in neurological disorders have investigated potential associations between blood or CSF damage marker levels and brain morphology/atrophy (Ayrignac et al., 2020; Barker et al., 2021; Deters et al., 2017; Illán-Gala et al., 2021; Plavina et al., 2020; Schönknecht et al., 2003; Shahim et al., 2020). In non-neurodegenerative neuropsychiatric disorders, only two known studies exist which have investigated these markers and their associations with global measures of brain volume (Andreou et al., 2021; Li et al., 2021). Our aim with the present study was to combine fine-grained vertex-wise (i.e. highly regional) investigations of CT changes in AN with the information obtained from three different brain-derived damage markers. A vertex-wise approach allows to detect local effects, which might not be visible when averaging across regions or the whole cortex.

Our hypotheses were that serum concentrations of brain damage markers (NF-L, tau protein, and GFAP) in acAN would be negatively associated with CT (i.e. higher marker levels go along with lower CT) and that changes in marker levels following partial weight restoration would contribute above and beyond increase in BMI to explain normalization of CT. To test whether the observed effects were specific to AN, we conducted a follow-up analysis examining potential associations between damage markers and CT in a large sample of healthy participants.

## Materials and methods

### Participants

Study data were obtained from 54 underweight female patients with acute AN diagnosed according to the Diagnostic and Statistical Manual of Mental Disorders (DSM-5; American Psychiatric Association, 2013; age range 12–24 years). Two acAN were excluded after quality control of the structural MRI (sMRI) data (see online Supplementary Information 1.4.2), resulting in a final sample of 52 acAN. The additional HC group consisted of 149 female participants (age range 12–23 years) of whom two were excluded after quality control (final sample of 147 HC participants). The sample partially overlaps with our previous studies (Bahnsen et al., 2022; Bernardoni et al., 2016; Hellerhoff et al., 2021; see online Supplementary Information 1.1 for Details). AN patients were studied in the acutely underweight state (acAN-TP1) not more than 96 h after admission to intensive treatment and reassessed after short-term partial weight restoration (acAN-TP2; after an a priori-defined minimum BMI increase of 10%; all patients in the present sample had a BMI increase >14%). Participants with AN underwent treatment in eating disorder programs at a tertiary care university hospital. 98% of the participants identified as European (details in online Supplementary Information 1.1). The authors assert that all procedures contributing to this work comply with the ethical standards of the relevant national and institutional committees on human experimentation and with the Helsinki Declaration of 1975, as revised in 2008. All participants (and the legal guardians of underage participants) gave written informed consent.

AN was diagnosed using a modified version of the German expert form of the Structured Interview for Anorexia and Bulimia Nervosa (SIAB-EX; Fichter & Quadflieg, 1999) and required a BMI < 17.5 kg/m^2^ (or below the 10th age percentile, if younger than 15.5 years). HC had to be of normal weight, eumenorrhoeic, and without any history of psychiatric illness. Several additional exclusion criteria were applied to both AN and HC participants, including psychotropic medication in the four weeks preceding study participation (except for selective serotonin reuptake inhibitors, which were allowed in AN participants, *n* = 1 in acAN-TP1 and *n* = 2 in acAN-TP2; online Supplementary Information 1.1).

### Clinical measures

Psychopathology was assessed using the SIAB-EX (Fichter & Quadflieg, 1999), the ‘drive for thinness’ and ‘body dissatisfaction’ scales from the German version of the Eating Disorder Inventory-2 (EDI-2; Paul & Thiel, 2005), and the German version of the Beck Depression Inventory-II (BDI-II; Hautzinger, Keller, & Kühner, 2009). IQ was estimated using age-appropriate versions of the Wechsler Intelligence Scales (online Supplementary Information 1.2). Age- and gender-corrected BMI standard deviation scores (BMI-SDS; Hemmelmann, Brose, Vens, Hebebrand, & Ziegler, 2010; Kromeyer-Hauschild *et al*. 2001) were calculated from the BMI.

### Blood sampling and Simoa analysis

Blood sampling directly before the MRI scan and determination of NF-L, tau protein, and GFAP levels were carried out as previously described (Doose et al., 2021; Hellerhoff et al., 2021; online Supplementary Information 1.3) using the digital Simoa™ Human Neurology 4-Plex A assay in combination with the Simoa™ HD-1 Analyzer (both Quanterix, Lexington, MA, USA).

### MRI acquisition and processing and CT estimation

MRI scanning took place between 8 and 9 a.m. Studies on the acute effect of food intake on brain damage marker concentrations are lacking, but all participants were studied after an overnight fast to minimize potential influence of acute food intake on the results. High-resolution three-dimensional T1-weighted structural scans were acquired on a 3 T scanner (Magnetom Trio, Siemens, Erlangen, Germany) using a rapid acquisition gradient echo (MP-RAGE) sequence with the same parameters as in our previous studies (Bahnsen et al., 2022; Bernardoni et al., 2016; online Supplementary Information 1.4.1).

Each participant's T1-weighted MP-RAGE images were first preprocessed in an automated manner with the FreeSurfer software suite (https://surfer.nmr.mgh.harvard.edu/; version 5.3; Dale, Fischl, & Sereno, 1999; Desikan *et al*. 2006; Fischl & Dale, 2000; Fischl, Sereno, & Dale, 1999; Fischl et al. 2002, 2004). Thereafter quality control (online Supplementary Information 1.4.2) was carried out as described in our previous studies (Bahnsen et al., 2022; Bernardoni et al., 2016; Reuter, Schmansky, Rosas, & Fischl, 2012). All images underwent further longitudinal preprocessing with the FreeSurfer longitudinal stream. CT was calculated at each vertex on the tessellated surface and smoothed using a Gaussian kernel with a full-width-at-half-maximum (FWHM) of 10 mm (online Supplementary Information 1.4.2).

### Statistical analyses

#### Clinical variables and brain damage marker levels

Due to slight violations of normal distribution, marker concentration values were log-transformed prior to analysis. Extreme outliers (>3 standard deviations (s.d.) from the mean of the respective diagnostic group and time point after logarithmization) were excluded from all analyses (online Supplementary Information 1.5.1). For the analysis of demographic variables, non-parametric tests (Wilcoxon rank sum tests or Wilcoxon signed rank tests) were used since not all variables were normally distributed. Group comparisons of log-transformed damage marker levels were conducted using Welch two sample t-tests (for one-sided acAN-TP1 *v.* HC comparisons, with correction of degrees of freedom to account for differences between groups in the variance) and matched sample t-tests (for one-sided acAN-TP1 *v.* acAN-TP2 comparisons). One-sided t-tests were chosen because based on existing literature, we expected to find higher marker levels in acAN-TP1 compared to HC and in acAN-TP1 compared to acAN-TP2 as a sign of brain damage (Gaetani et al., 2019; Hellerhoff et al., 2021; Kawata et al., 2016; Nilsson et al., 2019; Randall et al., 2013; Yang & Wang, 2015). False discovery rate (FDR; Benjamini & Hochberg, 1995) correction was applied to account for multiple testing. Analysis of demographic and clinical variables and of damage marker levels was carried out using the software R (version 3.6.3; R Core Team, 2020).

#### Cortical thickness

Following Bernardoni et al. (2016), CT in AN was modeled at each vertex of the cortical surface with a linear mixed-effect (LME) model (separately for each damage marker and for each hemisphere) as
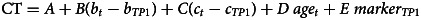
where *A* is the CT in acAN at the mean age and mean marker_TP1_ level, *b_t_* is BMI-SDS and *c_t_* is the log-transformed marker level (both at time *t*). *Age_t_* is the age at time *t* and *marker_TP1_* is the log-transformed marker-level in acAN-TP1 (both mean-subtracted) of the subject. B, C, D, and E thus represent the rate of CT change in AN associated with change in BMI-SDS, change in marker levels, age (as a control variable) and marker levels in acAN-TP1, respectively. In this model, *A* was a random effect and all other terms were fixed across participants.

Of note, changes in BMI-SDS and damage marker levels correlate (Hellerhoff et al., 2021). As we are mainly interested in the effect of change in damage markers on CT change above and beyond the effect of change predicted by BMI-SDS, the term c_t_-c_TP1_ was orthogonalized with respect to b_t_-b_TP1_. Further elaborations on the statistical model can be found in Bernardoni et al. (2016) For the practical implementation of the model, customized FreeSurfer LME Matlab tools (https://surfer.nmr.mgh.harvard.edu/fswiki/LinearMixedEffectsModels; Bernal-Rusiel, Greve, Reuter, Fischl, & Sabuncu, 2013*a*; Bernal-Rusiel, Reuter, Greve, Fischl, & Sabuncu, 2013*b*; Reuter *et al*. 2012) were used.

Since our analyses revealed an association between NF-L levels in acAN-TP1 and CT, we investigated whether this association was specific to acAN by assessing the same relationship in an independent sample of HC participants. Therefore, in a follow-up analysis we used a general linear model (GLM) to model CT in HC as
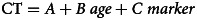
where *A* represents the CT in HC at the mean age and mean marker level and *age* is the mean-subtracted age of the subject (as a control variable), and *B* thus represents the effect of age on CT. *Marker* is the mean-subtracted log-transformed marker-level of the subject, and *C* thus represents the effect of marker-levels on CT. For the practical implementation we used FreeSurfer Matlab tools (https://surfer.nmr.mgh.harvard.edu/fswiki/FsTutorial/GroupAnalysis).

A supplementary model including both groups was built to locate areas with an effect of diagnostic group (acAN *v.* HC) on CT. This model is described in the online Supplementary Information (1.5.2).

## Results

### Study sample and clinical measures

To characterize our main AN sample in terms of clinical features and brain damage marker abnormalities, it is put in contrast with the independent HC sample used for the follow-up analysis in Table 1. AN and HC participants did not differ with regard to age or IQ. AcAN-TP1 participants had significantly lower BMI-SDS and minimal lifetime BMI and higher symptom levels than HC (EDI-2 and BDI-II). Group comparisons revealed significantly increased levels of NF-L, tau protein, and GFAP in acAN-TP1 compared to HC. As expected and shown in our previous studies (Bahnsen et al., 2022; Bernardoni et al., 2016), acAN-TP1 showed widespread reductions in CT (online Supplementary Fig. S1).

**Table 1.** Median values [interquartile range] of sample characteristics and damage marker levels and statistics of group comparisons

	acAN-TP1^a^		acAN-TP2^b^		HC		acAN-TP1 *v.* HC		acAN-TP1 *v.* acAN-TP2	
	Median [IQR]	*n*	Median [IQR]	*n*	Median [IQR]	*n*	W	*p*	V	*p*
Age (years)	16.4 [15.4; 17.3]	52	16.6 [15.6; 17.525]	52	16.7 [14.7; 18.35]	147	3484	0.3443	–	–
IQ	109 [101.5; 124]^c^			48	112 [107.25; 118]	146	3401.5	0.7622	–	–
BMI-SDS	−2.670 [−3.512; −2.082]	52	−0.529 [−0.943; −0.302]	52	−0.067 [−0.550; 0.321]	147	0	<0.001	0	<0.001
Minimal lifetime BMI	14.908 [14.134; 15.552]	52	–	–	19.375 [18.381; 20.849]	130	42	<0.001	–	–
EDI-2 Drive for thinness	27.5 [22; 36.25]	52	25 [17.75; 32.5]	51	13 [9; 16]	147	6842.5	<0.001	857	0.008
EDI-2 Body dissatisfaction	34 [27.75; 45]	52	37 [25; 45]	51	21 [16.5; 29]	147	6070	<0.001	604	0.536
BDI-II	23 [16.5; 32.25]	52	14 [7; 21]	51	3 [1.5; 7]	147	7125.5	<0.001	1184.5	<0.001

	Median [IQR]	*n*	Median [IQR]	*n*	Median [IQR]	*n*	*t*	*df*	*p*	Lower bound 95% CI	*t*	*df*	*p*	Lower bound 95% CI
NF-L^d^	10.4 [6.97; 14.90]	51	6.09 [4.770; 7.725]	51	6.11 [4.88; 7.35]	145	6.444	67.461	<0.001	0.164	10.436	50	<0.001	0.181
Tau protein^d^	1.39 [0.942; 1.685]	51	1.42 [1.070; 1.745]	51	1.08 [0.708; 1.380]	145	4.007	85.786	<0.001	0.073	0.004	50	0.498	−0.041
GFAP^d^	135.5 [108; 169.25]	52	107 [76.9; 131.0]	51	92.75 [77.10; 112.75]	146	5.715	68.914	<0.001	0.118	7.931	50	<0.001	0.089

*Note.* acAN-TP1, participants with acute anorexia nervosa; acAN-TP2, participants with anorexia nervosa after partial weight restoration; HC, healthy control participants; W, Test statistic of the Wilcoxon rank sum test with continuity correction; V, Test statistic of the Wilcoxon signed rank test with continuity correction; BMI-SDS, body mass index standard deviation score; BMI, body mass index; EDI-2, Eating Disorder Inventory, version 2; BDI-II, Beck Depression Inventory, version 2; NF-L, neurofilament light; GFAP, glial fibrillary acidic protein; CI, confidence interval of the group difference. *p*-values <0.05 were considered statistically significant and all significant group differences remained significant when applying false discovery rate correction for multiple comparisons (correction for eleven group comparisons of descriptive variables and for six group comparisons of marker values, respectively).

a 43 AN participants were of the restrictive and nine of the binge-eating/purging subtype.

b Mean duration between assessments in AN patients was 81.87 days (range 35–154).

c IQ was only measured once in AN participants after weight gain.

d For better interpretability, median and IQR of the raw marker values are displayed. However, group comparisons were computed with log-transformed marker values due to slight violations of normal distribution.

Between acAN-TP1 and acAN-TP2, BMI-SDS increased, while BDI-II scores and the EDI-2 subscale ‘Drive for thinness’ decreased. There was a decrease from acAN-TP1 to acAN-TP2 in NF-L and in GFAP levels, but not in tau protein levels (Table 1). As in our previous reports (Bahnsen et al., 2022; Bernardoni et al., 2016), with increasing BMI also CT increased in acAN-TP2 across many regions of the cerebral cortex (online Supplementary Fig. S2).

### Association of damage marker levels with CT

**Fig. 1.**
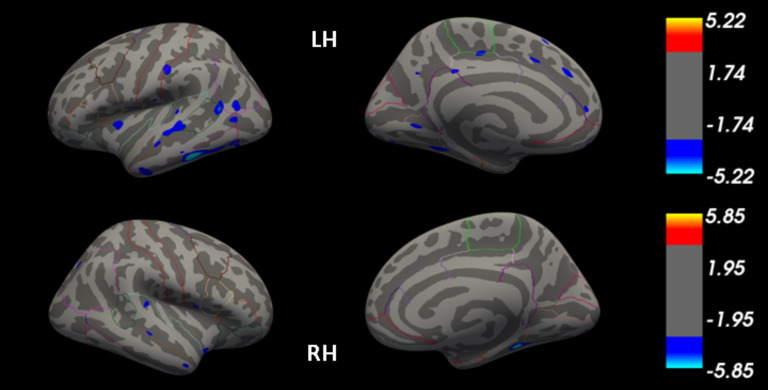
Association of neurofilament light (NF-L) levels with cortical thickness (CT) in patients with anorexia nervosa (AN). FDR-corrected statistical maps (*q* < 0.05) displaying regions in which log-transformed NF-L levels are associated with CT plotted on the inflated surface of a standard average subject. The color scale shows *p*-values expressed as –log10(p). Cool colors indicate a negative association between CT and NF-L. Abbreviations: LH, left hemisphere; RH, right hemisphere. Colored outlines correspond to anatomical labels of the Desikan-Killiany atlas (Desikan et al., 2006).

NF-L levels at TP1 were significantly associated with CT in acAN participants in several brain regions (Fig. 1) with prominent clusters located in the bilateral temporal lobe. Supplementary analyses revealed that these clusters were mainly located in areas where CT was decreased in acAN-TP1 compared with HC (online Supplementary Fig. S3). Neither tau protein nor GFAP levels at TP1 were associated with CT in acAN participants. Age in acAN was negatively associated (smaller CT at higher age) with CT in small clusters of the right hemisphere in the NF-L and tau models (online Supplementary Fig. S4). For none of the markers, change in protein levels predicted longitudinal change in CT (above and beyond change in BMI-SDS).

In the follow-up analysis with HC participants, we found the expected associations between age and CT. However, none of the damage markers was associated with CT in HC.

## Discussion

We tested whether elevated brain damage marker levels in AN are associated with CT. We found negative associations between NF-L (but not tau protein or GFAP) levels and CT in acAN in several brain regions with the most predominant clusters in regions of the bilateral temporal lobe (which showed particularly reduced CT relative to HC). This association was absent in HC. While elevated brain damage marker levels and reduced CT have been reported independently in non-neurodegenerative psychiatric illnesses (Bavato et al., 2021; Bernardoni et al., 2016; for the ENIGMA-Major Depressive Disorder Working Group et al., 2017; Hellerhoff et al., 2021; Nilsson et al., 2019), we show here for the first time a link between the two measures. Our findings suggest that the reductions in CT seen in AN might be at least partially related to axonal damage processes.

Studies in neurodegenerative diseases have suggested associations between elevated NF-L levels and global measures of brain volume (Barker et al., 2021; Cantó et al., 2019; Meeter et al., 2019; Plavina et al., 2020; Rajan et al., 2020). Studies conducted with local (voxel-/vertex-wise) measures of brain atrophy mostly confirmed these results. In Alzheimer's Disease, for instance, correlations between NF-L levels and CT have been found in the left and right lateral temporal lobes and in right inferior parietal and left superior frontal regions (Alcolea et al., 2017; Illán-Gala et al., 2021). In frontotemporal lobar degeneration related syndromes, local associations of NF-L levels with CT have been found in frontal, temporal, and parietal areas of both hemispheres (Alcolea et al., 2017; Falgàs et al., 2020; Illán-Gala et al., 2021; Spotorno et al., 2020). Therefore, while structural alterations in acAN seem more spatially homogeneous compared to neurodegenerative diseases [e.g. in Alzheimer's Disease the most pronounced reductions are located in the hippocampus and the temporal pole (Karow et al., 2010)], the neural mechanisms underlying these alterations might be similar, and seem in both cases related to axonal damage.

Regarding neuropsychiatric (non-degenerative) disorders, only one known study has investigated associations between NF-L and global measures of brain volume: In alcohol dependence, elevated levels of NF-L were found to be associated with the degree of WM lesions and were negatively correlated with global WM volume (Li et al., 2021). The present study is to our knowledge the first to simultaneously examine the relationship between three different brain damage markers and imaging measures of brain health in a neuropsychiatric disorder by means of a vertex-wise measure of brain alterations. Our results suggest that NF-L might be a marker of structural brain damage processes even in diseases that are not primarily caused by direct brain injury or neurodegeneration and that NF-L might potentially be a better marker for this purpose than tau protein or GFAP.

Speculatively, the differential relationships found between the three investigated markers and CT might be due to their different origins: NF-L is abundant in large-caliber myelinated axons (Gaetani et al., 2019; Zetterberg et al., 2013), while tau protein is predominantly expressed in thin non-myelinated axons of cortical interneurons (Trojanowski et al., 1989; Zetterberg et al., 2013). In line with this, a recent study found that CT reductions in AN are more prevalent in regions with greater structural and functional connectivity to other brain regions (‘hubs’; Bahnsen et al., 2022). Neuronal damage or remodeling processes occurring in the acutely underweight state of AN (Hellerhoff et al., 2021; King et al., 2018; Nilsson et al., 2019) might thus be more pronounced in these regions. In contrast, GFAP is released from astrocytes (Aurell et al., 1991; Eng et al., 2000) and regions characterized by a higher astrocyte gene expression (among others) seem to be less affected by AN-related CT reductions in human studies (Bahnsen et al., 2022). However, rodent studies support a role for astrocytes in models of AN (Frintrop et al., 2019).

Using a vertex-wise analysis approach, we were able to locate cortical regions, in which NF-L was associated with cortical thinning in AN. The largest clusters were located in the bilateral temporal lobe. Associations between NF-L and CT have previously been found in these regions in dementia (Alcolea et al., 2017; Illán-Gala et al., 2021). In the current study, the cluster observed in the right hemisphere was located mainly in the fusiform area and in the left hemisphere, the most prominent cluster was in the inferior temporal cortex. Reduced brain volume/CT of these areas in AN has been repeatedly reported (Bahnsen et al., 2022; Bernardoni et al., 2016; Brooks et al., 2011; Zhang et al., 2018) and also functional alterations have been observed (McAdams et al., 2016; Phillipou et al., 2015; Romero Frausto et al., 2021; Suda et al., 2013; Uher et al., 2005; Vocks, Herpertz, Rosenberger, Senf, & Gizewski, 2011).

Taken together with our finding, these results suggest that the temporal lobe, especially the right fusiform gyrus, might be particularly affected by neuronal-injury-related structural brain alterations occurring in acute AN. However, the present results only allow speculations about the reason why NF-L is associated with CT in exactly these temporal areas. Both the inferior temporal cortex and the fusiform gyrus play a role in visual tasks such as object, face, and body perception (Conway, 2018; Miyashita, 1993; Peelen & Downing, 2005; Weiner & Zilles, 2016). Alexithymic traits are a core feature of AN (Gramaglia, Gambaro, & Zeppegno, 2020) and some studies report altered brain activation patterns in AN in response to emotional face stimuli, e.g. in the fusiform gyrus (Fonville, Giampietro, Surguladze, Williams, & Tchanturia, 2014; Lulé, Müller, Fladung, Uttner, & Schulze, 2021) or increased activation in response to own face stimuli (McAdams et al., 2016; Phillipou et al., 2015). Also for body perception tasks, altered activation of the fusiform gyrus in AN has been reported (Suda et al., 2013; Uher et al., 2005). An alternative explanation could however be that the effect of axonal damage seen as an association of NF-L with CT is widespread (as is the effect of cortical thinning) and that because of relatively small effect sizes of NF-L elevations in AN we might have been able to observe it only in small clusters. Other small significant clusters, in which NF-L was associated with cortical thinning in AN, were found in several brain regions including e.g. frontal and parietal regions and the insula. However, due to the small extent of these clusters and their distributed localization, further studies replicating these results are needed to allow reliable interpretations of their exact localization.

Besides the contribution of the present results to hypothetical explanations of the neurobiological mechanisms underlying brain structural alterations in acute AN, they could potentially also help future research in identifying targets for the development of new therapeutic interventions. Such potential interventions could include neuroprotective pharmacological agents mitigating the drastic consequences of undernutrition on brain structure (Frank, 2020; Hellerhoff et al., 2021; Robertson et al., 2019). Another interesting future research target would be the question whether markers such as NF-L might be associated with the clinical outcome (e.g. with therapy-induced weight gain, cognitive functions, long term outcome etc.) in AN. In multiple sclerosis, for instance, NFL levels predict the progression of the disease and can predict treatment response (Kapoor et al., 2020). If similar effects were seen in AN, NF-L could have a potential to become a low cost and minimally invasive marker for how much the brain has been affected by this devastating illness. This in turn could be helpful in understanding consequences of the brain alterations e.g. on a cognitive level and integrating this knowledge into therapeutic concepts.

Our hypothesis of an association of the longitudinal decrease in NF-L levels with the increase in CT was not supported by the data. This might be because NF-L is released into the blood stream upon axonal injury (Gaetani et al., 2019) and is thus mostly considered a damage marker rather than a marker for repair processes. Also, tau protein as well as GFAP was not associated with CT in our sample. This finding, however, might also be due to missing statistical power, since the effect sizes of the increases in tau protein and GFAP in acAN-TP1 compared to HC were smaller than for NF-L.

The findings of our study have to be considered in the light of some limitations. First of all, our sample included only female, mostly adolescent participants of mainly European ethnicity. The effects seen might thus not generalize to adult, male, or more chronic patient populations or to other ethnicities. For example, the adolescent brain might be particularly vulnerable due to ongoing developmental effects and also the brain damage marker levels could be influenced by maturational processes such as ongoing myelination, synaptic pruning, etc. (Casey, Jones, & Hare, 2008; Giedd, 2008). It also has to be taken into account, that pubertal status might have influenced the results but was not controlled for. Future studies should assess pubertal stage where possible. Second, we measured damage marker levels peripherally in blood samples. Therefore, factors like the integrity of the blood-brain barrier could influence the measured marker concentrations (Nilsson et al., 2019). On the other hand, this method has the advantage of being less invasive than CSF sampling. Furthermore, we attempted to differentiate between axonal and astroglial damage processes by using three different protein markers.

In the present study, we found associations of serum concentrations of the neuro-axonal marker NF-L in acAN with CT in several regions of both brain hemispheres, predominantly in the bilateral temporal lobe. This finding, together with the recent results from virtual histology and connectivity analyses of CT changes (Bahnsen et al., 2022), amplifies our understanding of the biological mechanisms underlying the drastic structural brain changes in AN. Future research will elucidate whether NF-L might serve as a marker for monitoring brain alterations in acute AN potentially supporting the development of personalized therapeutic concepts.

## Supporting information

Hellerhoff et al. supplementary material Hellerhoff et al. supplementary material

## Supplementary material

The supplementary material for this article can be found at https://doi.org/10.1017/S0033291723000387.
