# Supplementary material for: Serum neurofilament light concentrations are associated with cortical thinning in anorexia nervosa
Source: Psychol Med. 2023 Mar 27;53(15):7053–61. doi: 10.1017/S0033291723000387 (PMC10719626; doi:10.1017/S0033291723000387)
Supplement: Hellerhoff et al. supplementary material [file S0033291723000387sup001.pdf]

Supplementary Information to

"Serum Neurofilament Light Concentrations are Associated with Cortical Thinning in Anorexia Nervosa"

Authors:

Inger Hellerhoff, MSc[1,2], Fabio Bernardoni, PhD[1], Klaas Bahnsen[1], Joseph A. King, PhD[1], Arne Doose, MSc[1], Sophie Pauligk, MSc[1], Friederike I. Tam, MD[1,2], Merle Mannigel[1], Katrin Gramatke, Dipl.-Psych.[3], Veit Roessner, MD[3], Katja Akgün, MD[4], Tjalf Ziemssen, MD[4], Stefan Ehrlich, MD, PhD[1,2,*]

[1]Translational Developmental Neuroscience Section, Division of Psychological and Social Medicine and Developmental Neuroscience, Faculty of Medicine, Technische Universität Dresden, Dresden, Germany

[2]Eating Disorder Research and Treatment Center, Department of Child and Adolescent Psychiatry, Faculty of Medicine, Technische Universität Dresden, Dresden, Germany

[3]Department of Child and Adolescent Psychiatry, Faculty of Medicine, University Hospital Carl Gustav Carus, Technische Universität Dresden, Dresden, Germany

[4]Center of Clinical Neuroscience, Neurological Clinic, Faculty of Medicine, University Hospital Carl Gustav Carus, Technische Universität Dresden, Dresden, Germany

*Correspondence to: S. Ehrlich, Technische Universität Dresden, Faculty of Medicine, University Hospital C. G. Carus, Dresden, Division of Psychological and Social Medicine and Developmental Neuroscience, Fetscherstraße 74, 01307 Dresden, Germany; Telephone: +49 351 4584099; Fax: +49 351 4585526; E-mail: transden.lab@uniklinikum-dresden.de

1. Materials and Methods

1.1 Participants

Healthy control participants (HC) were recruited through advertisement among middle school, high school and university students. Normal weight in HC was defined by a body mass index (BMI) between 18.5 kg/m^2 and 30 kg/m^2 (or between the 10th and the 97th age percentile, in participants younger than 18 years). Additional exclusion criteria for HC were a lifetime BMI below 17.5 kg/m^2 (or below the 10th age percentile when younger than 18 years) and substantial weight loss in the four weeks preceding study participation. Participants with anorexia nervosa (AN) were included if no substantial weight gain was reported in the four weeks preceding the first study point. In addition to the exclusion criteria mentioned in the main manuscript, participants in both groups were excluded if they had any history of bulimia nervosa or binge eating disorder, and any medical or neurological condition that might affect appetite, eating behavior, or body weight, any history of organic brain syndrome, dementia, schizophrenia, psychosis, or bipolar disorder. Further exclusion criteria for all participants were an IQ below 85, current substance abuse, current inflammatory, neurologic, or metabolic illness, clinically relevant anemia, pregnancy, and breast feeding. Information relevant to inclusion/exclusion criteria, including lifetime neurological disorders and possible confounding variables was obtained using the SIAB-EX (Fichter & Quadflieg, 1999), our own semi-structured interview, and medical records. Information about comorbid diagnoses in AN participants was obtained from medical records and confirmed by an expert clinician. Of the AN participants, 51 identified as European and one as Asian. In the HC group, 144 individuals identified as European, two as African and one as Asian.

Serum marker values of the complete AN sample and of a subgroup of the HC sample have been analyzed before (Doose et al., 2021; Hellerhoff et al., 2021) and the current structural magnetic resonance imaging (sMRI) sample partially overlaps with the sMRI data analyzed by Bernardoni et al. and Bahnsen et al. (Bahnsen et al., 2022; Bernardoni et al., 2016). The adequacy of the sample size for the analysis of cortical thickness (CT) was assured by consulting previous studies from our and other groups (Bernardoni et al., 2016; Cascino et al., 2020; King et al., 2015). Due to missing effect sizes

regarding neurofilament light (NF-L), tau protein, and glial fibrillary acidic protein (GFAP) levels in AN, studies on other neurological or psychiatric disorders were consulted to assure the adequacy of the sample size (Evered, Silbert, Scott, Zetterberg, & Blennow, 2018; Rohrer et al., 2016; Weston et al., 2017).Study data were managed using the secure, web-based electronic data capture tool REDCap (Harris et al., 2009).

1.2 Clinical measures

IQ was estimated using full or short versions of age-appropriate German versions of the Wechsler Intelligence Scales for adults or children (HAWIK-IV [Petermann & Petermann, 2007] in n = 97; WISC-IV [Petermann & Petermann, 2011] in n = 1; WISC-V [Petermann, 2017] in n = 2; WIE [von Aster et al., 2008] in n = 88; WAIS-IV [Petermann, 2012] in n = 6). Short versions were used in 151 participants. In five cases, IQ was missing. Missing clinical measures were not imputed.

1.3 Blood sampling and simoa analysis

Venous blood samples were collected into vacutainer tubes between 7 and 9 a.m. after an overnight fast. To yield blood serum, blood samples were left to clot for 30 min at 6–8°C and then centrifuged (800 x g for 15 min) in a pre-cooled (5°C) centrifuge. The samples were then aliquoted into pre-cooled Eppendorf Tubes® and stored at -80° C. Determination of NF-L, tau protein, and GFAP levels was carried out using the digital Simoa™ Human Neurology 4-Plex A assay in combination with the Simoa™ HD-1 Analyzer (both Quanterix, Lexington, MA, USA) following the manufacturer's instructions. All samples were measured in duplicates, and the mean of the two measurements was used for all statistical analyses (except in one case, where only a single measurement could be obtained). The mean coefficient of variation between duplicates was 6.6% (NF-L), 7.9% (tau protein), and 3.4% (GFAP), respectively.

1.4 MRI acquisition and processing

1.4.1 MRI acquisition

As in our previous studies (Bahnsen et al., 2022; Bernardoni et al., 2016), high-resolution three-dimensional T1-weighted structural scans were acquired on a 3T scanner (Magnetom Trio, Siemens, Erlangen, Germany) using a rapid acquisition gradient echo (MP-RAGE) sequence with the following parameters: 176 sagittal slices (1 mm thickness, no gap), TR = 1900 ms; TE = 2.26 ms; flip angle = 9°; voxel size = $1.0 \times 1.0 \times 1.0 mm^3$, FoV = $256 \times 224 mm^2$, bandwidth of 200 Hz/pixel).

1.4.2 Cortical thickness estimation

As in Bernardoni et al. (2016), we used standard FreeSurfer procedures to measure CT (Dale, Fischl, & Sereno, 1999; Desikan et al., 2006; Fischl & Dale, 2000; Fischl et al., 2002; Fischl, Sereno, & Dale, 1999; Fischl et al., 2004). Surface reconstruction was first performed for each hemisphere. The quality of the surface reconstruction and segmentation was assured by visual inspection by a trained examiner with the support of quality assurance tools implemented in FreeSurfer (https://surfer.nmr.mgh.harvard.edu/fswiki/QATools). All images underwent further longitudinal preprocessing with the FreeSurfer longitudinal stream. Specifically, the longitudinal pipeline first creates an unbiased within-subject template space and image (Reuter & Fischl, 2011) using robust, inverse consistent registration (Reuter, Rosas, & Fischl, 2010). Subsequently, several processing procedures are implemented including skull stripping, Talairach transformation and atlas registration in addition to generation of spherical surface maps and parcellations with common information from the within-subject template. The thickness at each vertex is computed as the average of two distances (Fischl & Dale, 2000; Greve & Fischl, 2018): the first is the distance from each white surface vertex to their corresponding closest point on the pial surface (not necessarily at a pial vertex); the second is the distance from the corresponding pial vertex to the closest point on the white surface (again, not necessarily at a vertex; Greve & Fischl, 2018). CT was smoothed using a Gaussian kernel with a full-width-at-half-maximum (FWHM) of 10 mm.

1.5 Statistical analyses

1.5 1 Outlier exclusion

Extreme outliers (> 3 standard deviations (SD) from the mean of the respective diagnostic group and time point after logarithmization) were excluded from all analyses resulting in the exclusion of four NF-L concentration values (*n* = 1 acAN-TP1, *n* = 1 acAN-TP2, and *n* = 2 HC), four tau protein concentration values (*n* = 1 acAN-TP1, *n* = 1 acAN-TP2, and *n* = 2 HC), and two GFAP concentration values (*n* = 1 acAN-TP2, and *n* = 1 HC).

1.5.2 Supplementary linear mixed effects model (LME)

To locate areas with an effect of study group (acAN vs. HC) on CT, we built a model including both acAN and HC participants. In this model, CT was estimated as

$$\mathrm{CT} = A + \Delta_{acAN} + B_{acAN}(b_t - b_{TP1}) + C\ age$$

A in this model is the CT in HC at the mean age (of the complete sample), *age* is the mean-subtracted age of the subject at the time of the scan, and *C* is the rate of age-related changes. b_t is BMI-SDS at time *t*. B_{acAN} is thus the rate of CT change associated with change in BMI-SDS. Δ_{acAN} is the difference in CT at mean age between acAN-TP1 and HC (because $(b_t - b_{TP1}) = 0$ when t = TP1).

2. Results

2.1 Supplementary linear mixed-effect (LME) Model

In the supplementary LME model including all participants, CT was strongly and mainly positively associated with change in BMI-SDS in both hemispheres. Age was negatively associated with CT in widespread clusters of both hemispheres, with exceptions in some small positive clusters in entorhinal, temporal, postcentral, and occipital regions. Study group was negatively associated with CT in widespread clusters of both hemispheres (thinner cortex in acAN compared to HC), with exceptions in some small positive clusters in entorhinal/temporal, precentral and anterior cingulate regions (Supplementary Figure S1).

2.2 Supplementary Figures

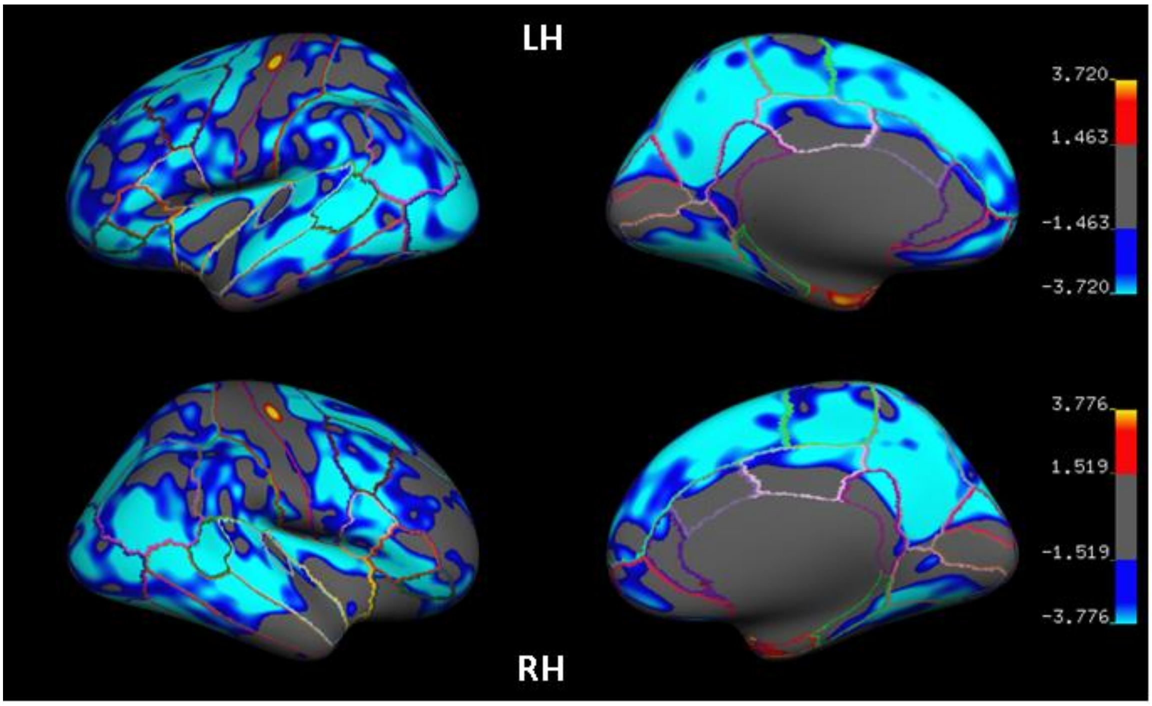

Supplementary Figure S1. Results of vertex-wise analysis of cortical thickness (CT) in acAN-TP1 patients relative to healthy controls (HC). FDR-corrected statistical maps ($q < .05$) displaying regions in which study group is associated with CT plotted on the inflated surface of a standard average subject. The color scale shows p-values expressed as –log10(p). Warm colors indicate an increased CT in patients with acute AN, cool colors indicate a decreased CT in patients with acute AN. Abbreviations: LH, left hemisphere; RH, right hemisphere. Colored outlines correspond to anatomical labels of the Desikan-Killiany atlas (Desikan et al., 2006).

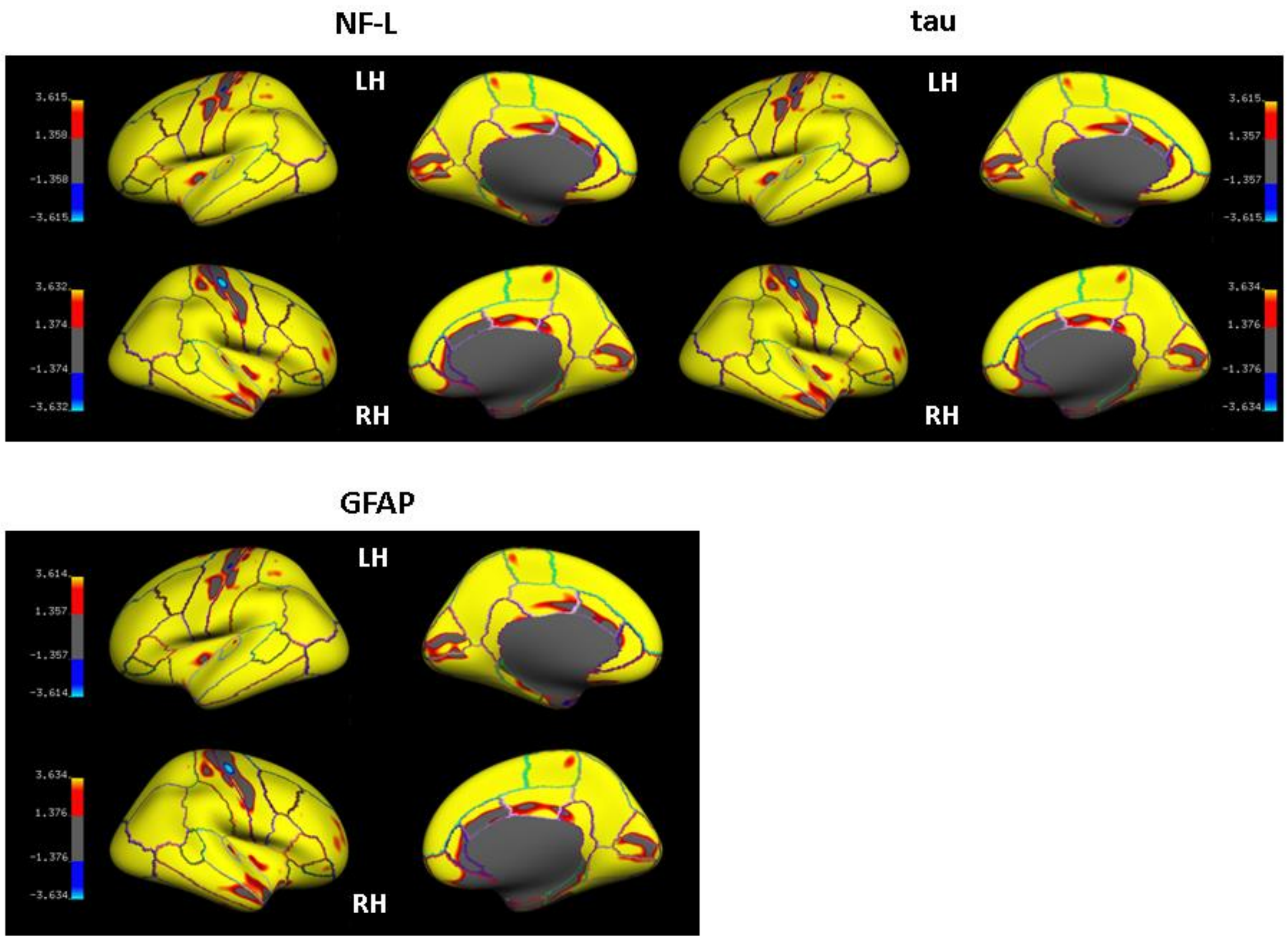

Supplementary Figure S2. Association of change in Body Mass Index (BMI-SDS) with cortical thickness (CT) in patients with anorexia nervosa (AN) after partial weight restoration. FDR-corrected statistical maps ($q < .05$) displaying regions in which change in BMI-SDS is associated with CT plotted on the inflated surface of a standard average subject. The color scale shows p-values expressed as $-\log10(p)$. Warm colors indicate an increased CT as a function of greater change in BMI-SDS. Abbreviations: LH, left hemisphere; RH, right hemisphere. Colored outlines correspond to anatomical labels of the Desikan-Killiany atlas (Desikan et al., 2006).

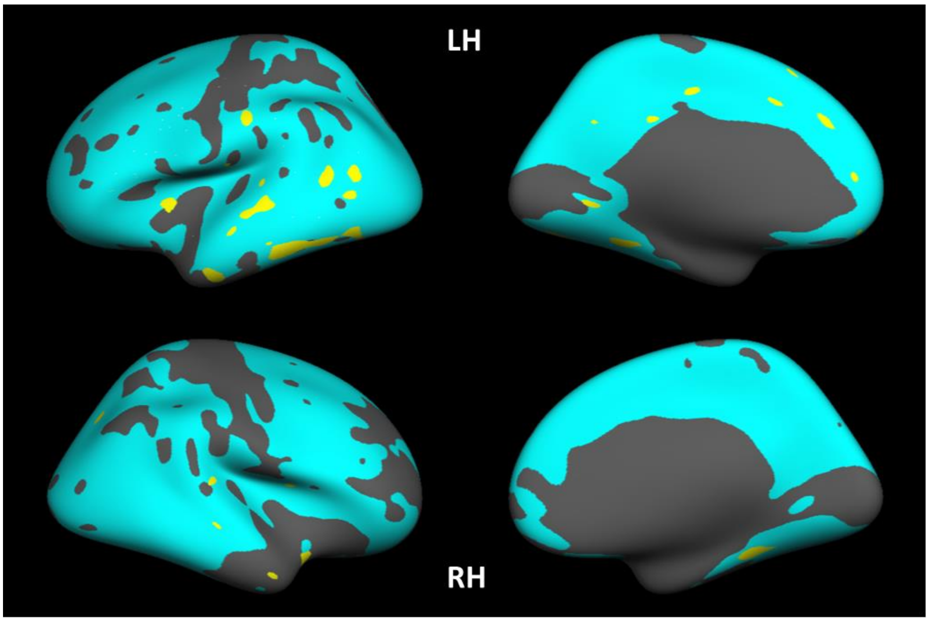

Supplementary Figure S3. Overlap between areas, in which cortical thickness (CT) in the supplementary analyses was reduced in patients with acute anorexia nervosa (acAN-TP1) (turquoise) and areas, in which CT in participants with anorexia nervosa was associated with neurofilament light levels in the main analyses (yellow). For better readability, the small clusters of increased CT in acAN-TP1 are not displayed in this image. Abbreviations: LH, left hemisphere; RH, right hemisphere.

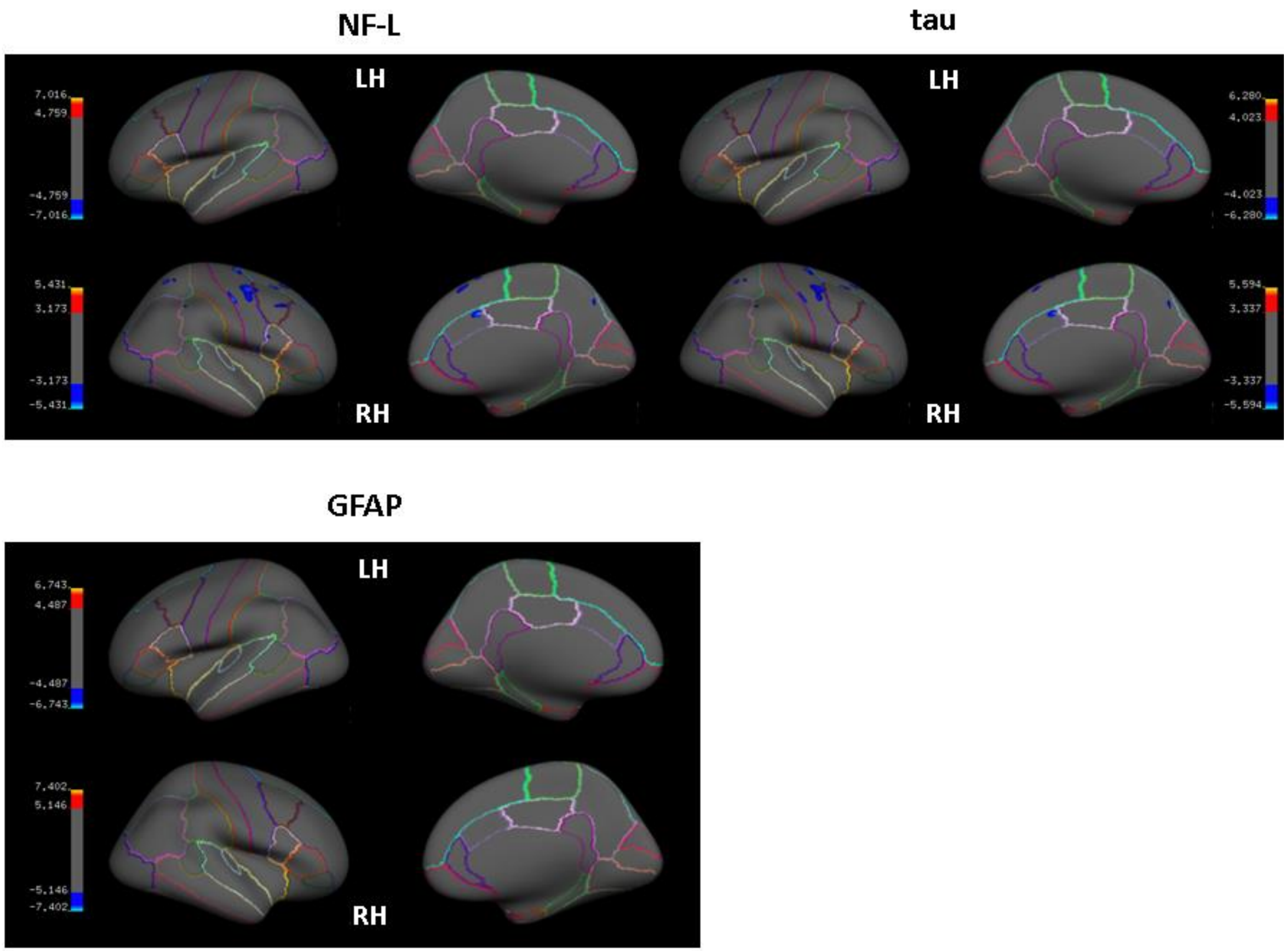

Supplementary Figure S4. Association of age with cortical thickness (CT) in patients with anorexia nervosa (AN). FDR-corrected statistical maps ($q < .05$) displaying regions in which (mean-subtracted) age is associated with CT plotted on the inflated surface of a standard average subject. The color scale shows p-values expressed as –log10(p). Cool colors indicate a decrease in CT as a function of higher age. Abbreviations: LH, left hemisphere; RH, right hemisphere. Colored outlines correspond to anatomical labels of the Desikan-Killiany atlas (Desikan et al., 2006).

References

Bahnsen, K., Bernardoni, F., King, J. A., Geisler, D., Weidner, K., Roessner, V., … Ehrlich, S. (2022). Dynamic Structural Brain Changes in Anorexia Nervosa: A Replication Study, Mega-analysis, and Virtual Histology Approach. *Journal of the American Academy of Child & Adolescent Psychiatry*, S0890856722001836. doi: 10.1016/j.jaac.2022.03.026

Bernardoni, F., King, J. A., Geisler, D., Stein, E., Jaite, C., Nätsch, D., … Ehrlich, S. (2016). Weight restoration therapy rapidly reverses cortical thinning in anorexia nervosa: A longitudinal study. *NeuroImage*, *130*, 214–222. doi: 10.1016/j.neuroimage.2016.02.003

Cascino, G., Canna, A., Monteleone, A. M., Russo, A. G., Prinster, A., Aiello, M., … Monteleone, P. (2020). Cortical thickness, local gyrification index and fractal dimensionality in people with acute and recovered Anorexia Nervosa and in people with Bulimia Nervosa. *Psychiatry Research. Neuroimaging*, *299*, 111069. doi: 10.1016/j.pscychresns.2020.111069

Dale, A. M., Fischl, B., & Sereno, M. I. (1999). Cortical surface-based analysis. I. Segmentation and surface reconstruction. *NeuroImage*, *9*(2), 179–194. doi: 10.1006/nimg.1998.0395

Desikan, R. S., Ségonne, F., Fischl, B., Quinn, B. T., Dickerson, B. C., Blacker, D., … Killiany, R. J. (2006). An automated labeling system for subdividing the human cerebral cortex on MRI scans into gyral based regions of interest. *NeuroImage*, *31*(3), 968–980. doi: 10.1016/j.neuroimage.2006.01.021

Doose, A., Hellerhoff, I., Tam, F. I., King, J. A., Seidel, M., Geisler, D., … Ehrlich, S. (2021). Neural and glial damage markers in women after long-term weight-recovery from anorexia nervosa. *Psychoneuroendocrinology*, *135*, 105576. doi: 10.1016/j.psyneuen.2021.105576

Evered, L., Silbert, B., Scott, D. A., Zetterberg, H., & Blennow, K. (2018). Association of Changes in Plasma Neurofilament Light and Tau Levels With Anesthesia and Surgery: Results From the CAPACITY and ARCADIAN Studies. *JAMA Neurology*, *75*(5), 542–547. doi: 10.1001/jamaneurol.2017.4913

Fichter, M., & Quadflieg, N. (1999). *SIAB. Strukturiertes Inventar für anorektische und bulimische Essstörungen nach DSM-IV und ICD-10*. Bern: Huber.

Fischl, B., & Dale, A. M. (2000). Measuring the thickness of the human cerebral cortex from magnetic resonance images. *Proceedings of the National Academy of Sciences of the United States of America*, *97*(20), 11050–11055. doi: 10.1073/pnas.200033797

Fischl, B., Salat, D. H., Busa, E., Albert, M., Dieterich, M., Haselgrove, C., … Dale, A. M. (2002). Whole brain segmentation: Automated labeling of neuroanatomical structures in the human brain. *Neuron*, *33*(3), 341–355. doi: 10.1016/s0896-6273(02)00569-x

Fischl, B., Sereno, M. I., & Dale, A. M. (1999). Cortical surface-based analysis. II: Inflation, flattening, and a surface-based coordinate system. *NeuroImage*, *9*(2), 195–207. doi: 10.1006/nimg.1998.0396

Fischl, B., van der Kouwe, A., Destrieux, C., Halgren, E., Ségonne, F., Salat, D. H., … Dale, A. M. (2004). Automatically parcellating the human cerebral cortex. *Cerebral Cortex*, *14*(1), 11–22. doi: 10.1093/cercor/bhg087

Greve, D. N., & Fischl, B. (2018). False positive rates in surface-based anatomical analysis. *NeuroImage*, *171*, 6–14. doi: 10.1016/j.neuroimage.2017.12.072

Harris, P. A., Taylor, R., Thielke, R., Payne, J., Gonzalez, N., & Conde, J. G. (2009). Research electronic data capture (REDCap)—A metadata-driven methodology and workflow process for providing translational research informatics support. *Journal of Biomedical Informatics*, *42*(2), 377–381. doi: 10.1016/j.jbi.2008.08.010

Hellerhoff, I., King, J. A., Tam, F. I., Pauligk, S., Seidel, M., Geisler, D., … Ehrlich, S. (2021). Differential longitudinal changes of neuronal and glial damage markers in anorexia nervosa after partial weight restoration. *Translational Psychiatry*, *11*(86). doi: 10.1038/s41398-021-01209-w

King, J. A., Geisler, D., Ritschel, F., Boehm, I., Seidel, M., Roschinski, B., … Ehrlich, S. (2015). Global cortical thinning in acute anorexia nervosa normalizes following long-term weight restoration. *Biological Psychiatry*, *77*(7), 624–632. doi: 10.1016/j.biopsych.2014.09.005

Petermann, F. (2012). *WAIS-IV. Wechsler adult intelligence scale* (2.). Frankfurt: Pearson Assessment & Information.

Petermann, F. (2017). *WISC-V. Wechsler Intelligence Scale for Children—Fifth Edition*. Frankfurt: Pearson Assessment & Information.

Petermann, F., & Petermann, U. (2007). *HAWIK-IV. Hamburg-Wechsler-Intelligenztest für Kinder IV* (1.). Bern: Huber.

Petermann, F., & Petermann, U. (2011). *WISC-IV. Wechsler Intelligence Scale for Children—Fourth Edition*. Frankfurt: Pearson Assessment & Information.

Reuter, M., & Fischl, B. (2011). Avoiding asymmetry-induced bias in longitudinal image processing. *NeuroImage*, *57*(1), 19–21. doi: 10.1016/j.neuroimage.2011.02.076

Reuter, M., Rosas, H. D., & Fischl, B. (2010). Highly accurate inverse consistent registration: A robust approach. *NeuroImage*, *53*(4), 1181–1196. doi: 10.1016/j.neuroimage.2010.07.020

Rohrer, J. D., Woollacott, I. O. C., Dick, K. M., Brotherhood, E., Gordon, E., Fellows, A., … Zetterberg, H. (2016). Serum neurofilament light chain protein is a measure of disease intensity in frontotemporal dementia. *Neurology*, *87*(13), 1329–1336. doi: 10.1212/WNL.0000000000003154

von Aster, M., Neubauer, A., & Horn, R. (2008). *WIE. Wechsler Intelligenztest für Erwachsene* (2.). Frankfurt: Pearson Assessment & Information.

Weston, P. S. J., Poole, T., Ryan, N. S., Nair, A., Liang, Y., Macpherson, K., … Fox, N. C. (2017). Serum neurofilament light in familial Alzheimer disease: A marker of early neurodegeneration. *Neurology*, *89*(21), 2167–2175. doi: 10.1212/WNL.0000000000004667
